# Testing the Reliability of Anchoring Susceptibility Scores

**DOI:** 10.5964/ejop.9891

**Published:** 2025-02-28

**Authors:** Lucia Weber, Lukas Röseler

**Affiliations:** 1 Department of Personality Psychology and Psychological Assessment, University of Bamberg, Bamberg, Germany; 2 Münster Center for Open Science, University of Münster, Münster, Germany; Dublin City University, Dublin, Ireland

**Keywords:** anchoring, anchoring effect, anchoring scores, anchoring strength, reliability

## Abstract

Whereas anchoring is a very robust and well-known effect that refers to the assimilation of numeric estimates toward previously considered numbers, the psychological mechanisms behind it have yet to be fully clarified. Research on theories on how susceptibility to anchoring is related to other personality parameters has not been able to provide sufficient empirical evidence of such relationships. A probable explanation is that anchoring scores lack reliability in most anchoring experiments. The present research examined whether reliability depends on the type of score used to capture anchoring susceptibility. In a classical anchoring experiment, men and women aged between 14 and 67 years (*N* = 78) were asked to estimate the true values of certain numbers (e.g., height of the Zugspitze mountain) after being confronted with either a high or a low anchor number. Four different anchoring scores that are commonly used to measure susceptibility to anchoring in anchoring research were computed for every person, as well as the scores’ reliabilities. The number and types of items were chosen to allow for reliable and valid measurement. Anchoring effects were present, but the reliabilities of all four scores were either very low or zero. These results reinforce the reliability problem that was also described by previous research. So far, there are no conditions under which anchoring susceptibility can be measured reliably, suggesting the development of new measures or even questioning the existence of individual differences in susceptibility to anchoring. In further research, other person-independent factors that may influence anchoring strength should be investigated to develop theories that can explain the psychological mechanisms behind anchoring.

Anchoring describes the phenomenon that people are influenced by a previously considered number when making a numerical estimate. In studies on the anchoring effect, people are initially confronted with an anchor number—often by having to respond to a comparative question, such as “Is the height of Mount Everest larger or smaller than 5,000 meters?” After answering this first question, when asked to give an actual estimate, people tend to assimilate their answer toward the anchor.

It has been proposed that people differ in the extent to which they are susceptible to this effect, depending on certain individual differences, such as general knowledge, cognitive abilities (e.g., Bergman et al., 2010), or the Big Five (e.g., McElroy & Dowd, 2007). The implicit assumption that susceptibility to anchoring is a person parameter can be found at the core of anchoring models, which aim to clarify which psychological mechanisms underlie anchoring effects, for example, in the Insufficient Adjustment Model (Epley & Gilovich, 2001).

In order to confirm and expand on these theories, considerable research effort has been put toward finding evidence for the moderating effects of personality traits on anchoring susceptibility—resulting in very mixed findings. Although significant effects have been found in many cases (Bergman et al., 2010; Cheek & Norem, 2022; Epley & Gilovich, 2006; Eroglu & Croxton, 2010; McElroy & Dowd, 2007; Teovanović, 2019), replication attempts have often failed (Cheek & Norem, 2019; Furnham et al., 2012; Stanovich & West, 2008) and meta-analyses have shown that for numerous moderators of personality, the average effect sizes are indistinguishable from zero (Röseler, 2021).

A probable reason for this pattern of results is that anchoring susceptibility has not been measured reliably in these studies, making it impossible to find significant correlations with other constructs. This *reliability problem* as an explanation for unclear findings was first described by Röseler et al. (2019) and was then further explained and backed up by an extensive amount of evidence, showing very low reliabilities for measures of anchoring susceptibility in a large proportion of personality moderator research on anchoring (Röseler, 2021; Schindler et al., 2021). These findings raise questions about the conditions under which anchoring susceptibility can be measured reliably or whether it cannot be measured reliably at all.

To answer these questions, the Open Anchoring Quest (Röseler et al., 2022; Röseler et al., 2023) was created. In this project, data from as many existing anchoring experiments as possible are being meta-analytically aggregated into one large data set. The methodology in the included studies varies to a great extent, such as in how the anchoring task is implemented, or in other parameters, such as sample size. The OpAQ data set makes it possible to systematically investigate the influence of a range of these factors on the reliability of anchoring susceptibility measurements.

One factor that has varied across studies is how the score depicting susceptibility to anchoring was calculated. Whereas the basis for this score is the difference between the true value of the item and the anchor value in all cases, there are various ways in which this difference has been converted into a score. These differences could be crucial for explaining different outcomes in the reliability. As the structure of the OpAQ data set is trial-based, it provides the opportunity to calculate the different anchoring susceptibility scores by using data from all the studies included in the data set. So far, evaluations have indicated that different ways of computing anchoring susceptibility actually lead to scores with different reliabilities.

In the present research, we examined this assumption in a classical anchoring experiment where people make estimates about certain measurements (e.g., the height of a mountain) after being confronted with either a high or a low anchor. Most studies in the OpAQ data set have used a small number of items, which makes it difficult to have reliable anchoring scores. These items are also oftentimes very homogeneous (e.g., only estimates of the lengths of rivers), so the validity of anchoring susceptibility is also questionable, as it can be confounded with a person’s ability to give correct estimates in a certain area of knowledge. To allow for reliable and valid measurements of anchoring susceptibility in the present research, we used a large number of heterogeneous items, which varied in the size of their true values, the unit of measurement, and area of knowledge.

To examine susceptibility to anchoring, it is also necessary for the anchors to have an actual effect on the estimates, that is, low anchors must lead to lower estimates, and vice versa. Therefore, our first hypothesis was that there would be an anchoring effect for at least two thirds of the items (anchoring hypothesis). Furthermore, for every participant, we computed four different anchoring susceptibility scores in order to compare their reliabilities afterwards. Based on the previous explanations, the second hypothesis was that at least one of these scores would have a different reliability than the others (reliability hypothesis). This study furthermore contributes to the OpAQ data set by providing data from a study that was specifically designed to test for reliability.

## Method

### Power Analysis

A power analysis was computed to determine the required sample size (the code is available at Weber & Röseler, 2022). Given α = β = 5%, average correlations between the scores as computed on the basis of the OpAQ data set (Röseler et al., 2022; version from August 27, 2021), and all Cronbach’s alphas = 0 except for one, which is at least .5, at least 79 participants were required.

### Planned Sample

Participants were recruited via *SurveyCircle* and *SurveySwap*, which are platforms that were specifically created for finding survey participants. They include a reward system in which a person can obtain points by participating in other researchers’ surveys. The more points one collects, the more participants one gains for one’s own study. Aside from gaining points, participants were given feedback on the extent of their susceptibility to anchoring effects as well as on the correctness of their answers to the survey, which was promoted as a general knowledge quiz. Before participating, they were also informed that the study is conducted for research about anchoring effects. There were no preselection rules; only access to the online survey was required for participation.

### Sample

After data curation, 78 out of the original 96 participants remained. The planned sample size of *N* = 79 was not achieved because one of the participants later turned out to have completed the questionnaire two times without having admitted to doing so in the control question; therefore, the second trial had to be excluded manually after the initial data curation. Achieving a sample size of *N* = 78 instead of the planned *N* = 79 led to a minor decrease in power from *T* = .95 to *T* = .94.

The sample was predominantly female (67.9%), and participants’ ages ranged from 14 to 67 years (*M* = 28.05, *SD* = 9.01). Underaged participants were not excluded from our research to improve representativeness and because the contents of the study can be rated ethically safe as it was also approved by the ethics committee of the University of Bamberg. Overall, 76 participants came from SurveyCircle and two participants came from SurveySwap.

### Materials and Procedure

#### Construction of Anchoring Items

The main components of the study were 18 anchoring items, for which estimates had to be made about the true values of certain measurements (e.g., the length of a river; see Table 1). The items were created with a focus on heterogeneity in the area of knowledge, the unit of measurement, and the magnitude of the true value. For each item, a low and a high anchor were constructed by multiplying and dividing the true value by a factor of 1.8, respectively.

**Table 1:** Anchoring Items With True Values, Anchors, and Effect Sizes

			Anchor			
Item	Unit	True Value	Form A	Form B	Cohen’s *d*	95% Confidence Interval
Length of the Danube	km	2,857	1,587	5,143	-0.32	[-0.79, 0.14]
Elon Musk's income per hour	mil. $	15	8	27	0.60	[0.09, 1.10]^a^
Average weight of a male polar bear	kg	450	810	250	0.92	[0.42, 1.42]^a^
Average temperature in June in Germany	°C	15.5	9	28	1.45	[0.86, 2.02]^a^
Size of a standard football field	sq km	7,140	3,967	12,852	0.07	[-0.38, 0.53]
Percentage of smokers in Germany	%	28	50	16	1.67	[1.06, 2.27]^a^
Birth year of Alexander the Great^b^	BC	336	187	605	0.46	[-0.06, 0.97]
Circumference of the Earth	km	40,075	72,135	22,264	0.63	[0.10, 1.15]^ a^
Average height of a woman in Germany	cm	163.5	91	294	0.62	[0.06, 1.17]^a^
Top speed of an ICE 4^c^	km/h	330	594	183	0.97	[0.43, 1.49]^a^
Height of the Zugspitze	m	2,962	5,332	1,646	0.64	[0.16, 1.11]^a^
Duration of the moon’s orbit around the earth	days	27	49	15	1.22	[0.66, 1.77]^a^
Average price of a new car in Germany	€	34,000	18,889	61,200	-0.26	[-0.73, 0.21]
Number of sugar cubes in one bottle of Coca Cola (0.33 l)	—	12	22	7	1.12	[0.60, 1.64]^a^
Number of manned moon landings so far	—	6	3	11	1.39	[0.79, 1.98]^a^
Shortest distance between Russia and the US	km	4	2	7	1.24	[0.70, 1.78]^a^
Age of the oldest human on earth	years	122	220	68	1.38	[0.75, 1.99]^a^
Chance of getting four correct numbers in the lottery	1 to …	1,147	2,065	637	0.89	[0.39, 1.37]^a^

*Note*. Larger values of Cohen’s *d* represent stronger anchoring effects (high anchor – low anchor).

^a^Effect size is significantly larger than 0 (i.e., *p* < .050, items where anchoring effects were present). ^b^The item *Birth year of Alexander the Great* was excluded before the statistical analysis. ^c^ICE stands for Intercity Express which is a system of high-speed trains in Germany.

#### Structure of the Questionnaire

The anchor study was carried out as an online questionnaire on *SoSciSurvey* (Leiner, 2019). The components of the questionnaire were as follows: On a start page, the participants were informed about the content of the quiz and the structure of the questions. Also, anchoring effects were explained. The participants were asked to answer the questions to the best of their ability and not to be influenced by the anchors presented to them. On the next page, demographic information regarding age and gender were requested. Afterwards, an individual code that was required for the person to receive individual feedback was created. Then, the actual “quiz” began, containing 18 anchoring items with either high or low anchors. Each item required a response to a comparative question (e.g., “Is the shortest distance between Russia and the US shorter or longer than 2 km?”) before the estimate was given to guarantee that the participants consciously perceived the anchor. After participants gave their answer and continued, their answer was presented to them again, and they had to give an estimate of the true value (e.g., “You have answered that the shortest distance between Russia and the US is longer than 2 km. How long do you think it actually is?”). The estimates were entered into an open textbox, with the unit already given. At the end of the questionnaire, a few follow-up questions were presented. For every anchoring item, the participants had to specify their prior knowledge about the true value of each item on a closed scale: “Did you (a) know, (b) estimate, or (c) guess the true value?” Two questions recorded the extent of the participants’ motivation to (a) give the best estimate they could give and (b) avoid being influenced by the anchors. Finally, two more questions were used to check whether the participants used external sources to answer the questions or took the quiz more than once. On the final page, the participants were thanked for completing the questionnaire and were provided with a link to the feedback page where they could see the correct answers to the anchoring items next to their own answers as well as their anchor susceptibility score compared with the other participants.

There were two parallel forms of the questionnaire with different anchor values. Each form had nine low and nine high anchors. The means of the anchors and the true values were approximately equal for the two forms. The form was randomly assigned when participants clicked on the link to the online study.

### Analysis Plan and Details About the Preregistration

To examine whether there was an anchoring effect for at least two thirds of the items (anchoring hypothesis), a between-subjects analysis was applied. For every anchoring item, a one-tailed *t* test of the difference between the estimates with low versus high anchors was computed (direction: larger estimates for high anchors than for low anchors).

To check for whether at least one of the anchoring susceptibility scores had a different reliability than the others (reliability hypothesis), a within-subjects analysis was applied. Four different anchoring susceptibility scores were computed for every participant and item. The scores were as follows:

1. Adjustment: difference between estimate and anchor.
2. Absolute adjustment: absolute difference between estimate and anchor.
3. 0–1 score: difference between estimate and anchor divided by difference between true value and anchor.
4. Restricted 0–1 score: the above score but with cut-offs at 0 and 1.

We tested whether the reliabilities of the four scores differed from each other by computing a chi-square test that compared the z-standardized Cronbach’s alphas. The significance criterion was set to .05 for both the *t* test and the chi-square test.

The study had been preregistered before the data were collected (Weber et al., 2021). Statistical analyses were computed with R version 4.0.2 (R Core Team, 2020) with the packages cocron (Diedenhofen & Musch, 2016) and ggplot2 (Wickham, 2016) and Microsoft Excel Version 2203 (Microsoft Corporation, 2022).

### Data Processing

One of the 18 anchoring items (birth year of Alexander the Great) had to be omitted due to an error in the specification of the unit in the questionnaire. Estimates within anchoring items were excluded if, (a) participants stated that they had already known the correct answers, or (b) the estimates were more than three standard deviations away from the mean value. After applying the item-based exclusion criteria, participants who used external sources when answering the anchoring items along with participants who had nine or fewer valid items were excluded from the data set. If participants completed the survey more than once, we included only their first one.

## Results

### Hypothesis Tests

There were significant anchoring effects for 14 of the remaining 17 items, meaning that high anchors resulted in higher estimates than low anchors. Among the items with significant anchoring effects, Cohen’s *d* ranged from 0.46 to 1.67 (see Table 1). Therefore, the data were consistent with the anchoring hypothesis, indicating that the anchors generally did influence the estimates in the expected way.

**Figure 1:**
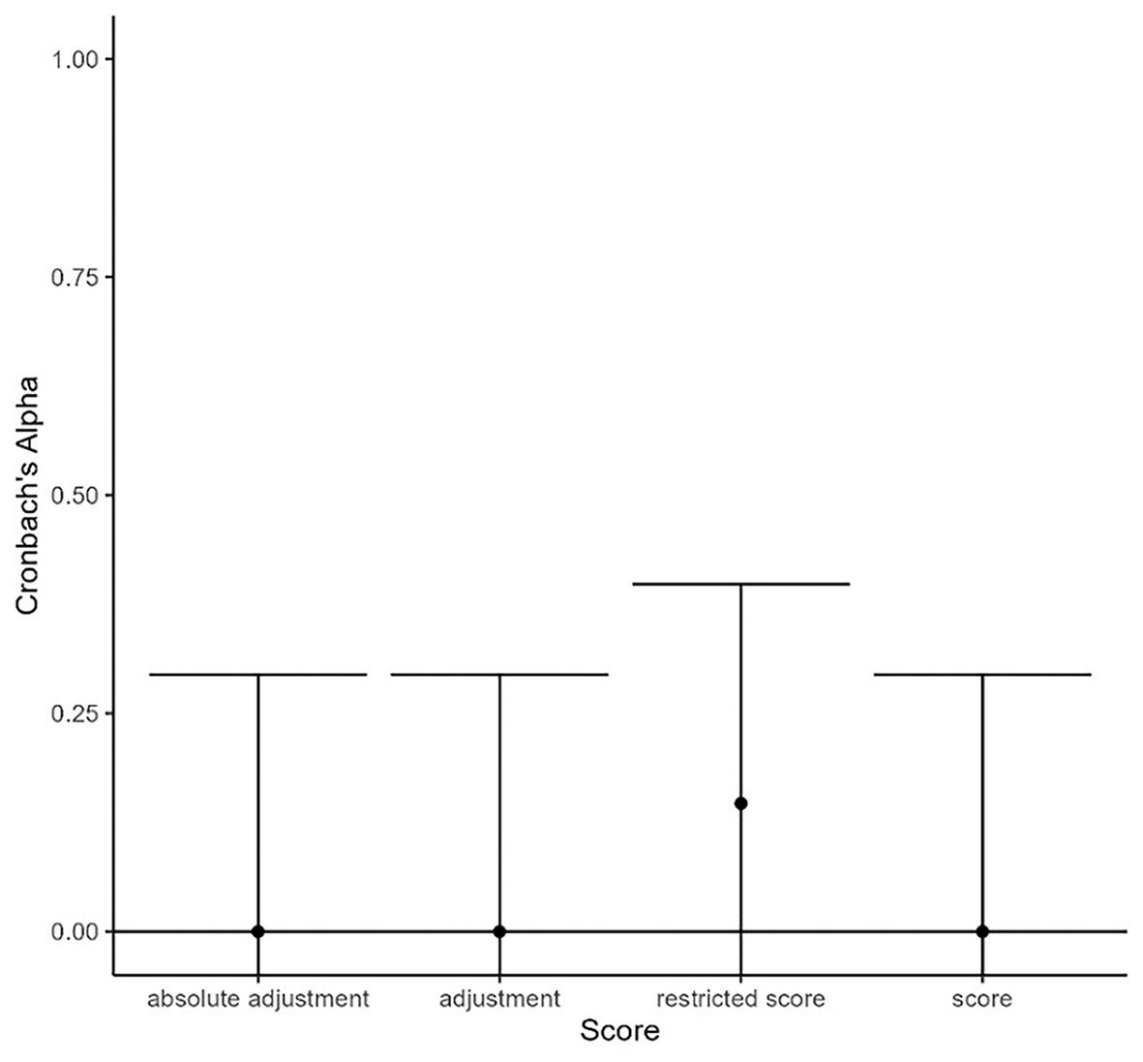
Reliabilities of the Anchoring Susceptibility Scores *Note*. Error bars represent 95% confidence intervals.

The reliabilities of the four anchoring susceptibility scores did not differ significantly from one another, χ^2^(3, *N* = 78) = 0.91, *p* = .824. Therefore, the data were not consistent with the reliability hypothesis. The Cronbach’s alpha values were set to zero for three of the scores because they had negative values. Only the restricted 0–1 score showed a small positive reliability (α = .15). Figure 1 illustrates these results.

### Exploratory Tests

Additional exploratory analyses were computed to investigate the influence of prior knowledge and motivation on the anchoring scores.

#### Prior Knowledge

To determine whether anchoring strength depends on the extent of prior knowledge, for every item, participants were grouped according to whether they guessed or estimated the true value. Mean absolute adjustment scores and effect sizes were then computed for the two groups. Table 2 presents the results.

**Table 2:** Mean Absolute Adjustment Score Depending on Prior Knowledge for Every Item

	Guessed		Estimated			
Item	*N*	*Mean (SD)*	*N*	*Mean (SD)*	Cohen’s *d*	[95% CI]
Length of the Danube	42	1,498.88 (1,531.25)	34	1,436.41 (1,171.20)	0.05	[-0.41. 0.50]
Elon Musk's income per hour	27	7.48 (4.94)	44	7.82 (6.22)	-0.06	[-0.54. 0.42]
Average weight of a male polar bear	24	243.96 (165.14)	46	201.41 (161.93)	0.26	[-0.24. 0.76]
Average temperature in June in Germany	48	14.06 (8.99)	27	13.22 (26.79)	0.05	[-0.42. 0.52]
Size of a standard football field	35	3,358.91 (3,257.33)	39	4,416.90 (3,107.05)	-0.33	[-0.79. 0.13]
Percentage of smokers in Germany	27	17.44 (10.52)	35	17.11 (7.36)	0.04	[-0.47. 0.54]
Birth year of Alexander the Great	52	370.08 (330.79)	23	327.30 (285.71)	0.14	[-0.36. 0.63]
Circumference of the Earth	30	18,535.53 (15,124.52)	48	17,478.69 (14,131.27)	0.07	[-0.38. 0.53]
Average height of a woman in Germany	3	93.33 (30.92)	53	97.70 (32.67)	-0.13	[-1.30. 1.03]
Top speed of an ICE 4	15	119.80 (120.06)	53	183.34 (118.67)	-0.53	[-1.11. 0.05]
Height of the Zugspitze	35	873.17 (826.10)	32	1,619.13 (982.35)	-0.83	[-1.32. -0.33]^a^
Duration of the moon’s orbit around the earth	11	7.09 (5.47)	62	6.58 (3.66)	0.13	[-0.51. 0.77]
Average price of a new car in Germany	27	20,316.52 (21,531.29)	35	18,705.20 (12963.34)	0.09	[-0.41. 0.60]
Number of sugar cubes in one bottle of Coca Cola (0.33 l)	27	10.04 (7.74)	49	16.78 (11.10)	-0.67	[-1.15. -0.19]^a^
Number of manned moon landings so far	39	4.59 (3.35)	28	5.04 (3.97)	-0.12	[-0.61. 0.36]
Shortest distance between Russia and the US	39	2,076.92 (3,639.81)	35	2,193.31 (3922.04)	-0.03	[-0.49. 0.43]
Age of the oldest human on earth	8	60.25 (36.47)	56	74.54 (25.62)	-0.53	[-1.27. 0.22]
Probability of getting four correct numbers in the lottery	38	294,200,127.42 (1,783,835,846.48)	34	28,723,127.32 (112,022,198.82)	0.20	[-0.26. 0.67]

*Note*.^a^Effect size is significantly larger than 0 (i.e., *p* < .050, items where estimates between the two groups differed).

#### Motivation

**Figure 2:**
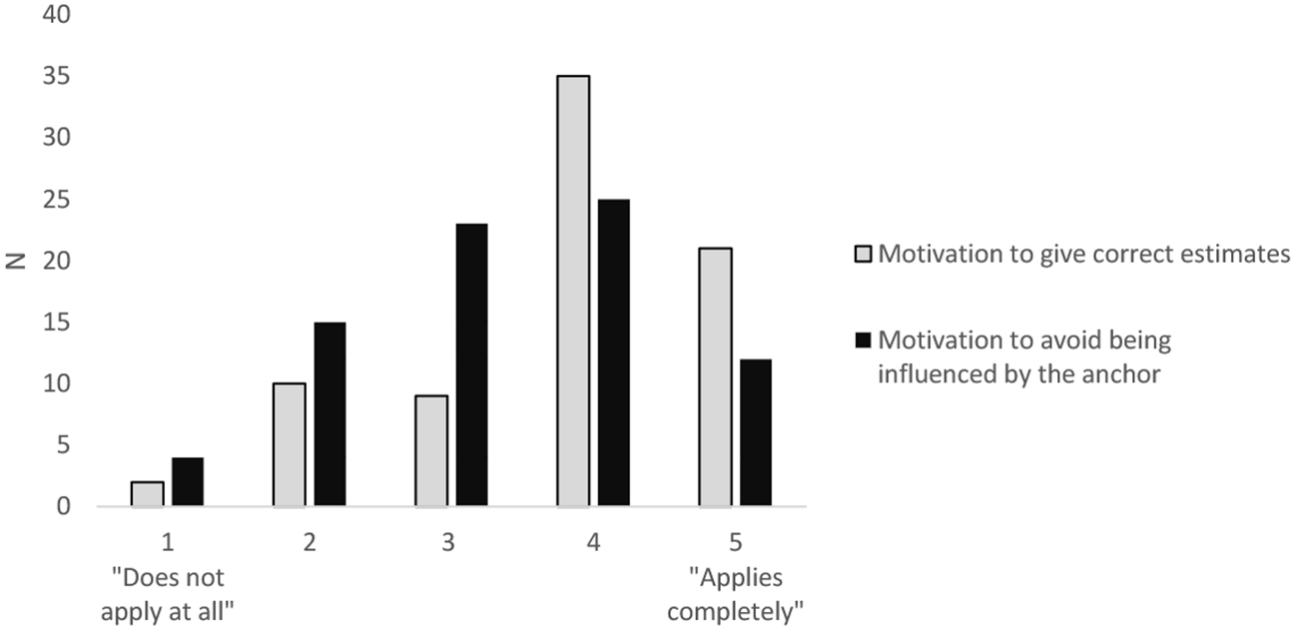
Descriptive Analysis of Participants’ Motivation When Completing the Anchoring Task

To get an overview of participants’ general level of motivation while completing the anchoring task, we analyzed the answers to the two items on motivation. The frequencies are depicted in Figure 2. As we were interested in whether higher motivation would lead to a higher reliability for anchoring scores, the Spearman correlation between the reliabilities of two anchoring scores (the absolute adjustment score and 0–1 score) and the answers to the motivation items were computed (Table 3).

**Table 3:** Reliabilities of Anchoring Measurements in Relation to Participants’ Motivation

	Reliability	
Motivation Item	Absolute adjustment	0–1 score
Motivation to give correct estimates	.07	-.15
Motivation to avoid being influenced by the anchor	.29	-.24

## Discussion

The present study was designed to investigate the reliabilities of different anchoring scores in order to clarify whether and under which conditions susceptibility to anchoring can be measured reliably.

### Anchoring Effect

Anchoring effects were found in 14 out of 18 items as shown by the fact that lower estimates were given when a low anchor was presented and vice versa. Therefore, the data were consistent with the anchoring hypothesis, meaning that the intended manipulation worked and thus laid the foundation for further examination of the anchoring measurements.

What is also worth noting is that participants were informed about anchoring effects before performing the task. Not only was the effect explained to them, but they were also asked to avoid being influenced by the anchor when giving their estimates. The anchoring manipulation worked well anyway, which again underscores the robustness of anchoring effects.

### Anchoring Susceptibility

Because there were no significant differences in the reliabilities of the four anchoring susceptibility scores, the reliability hypothesis was rejected. In fact, the most reliable score, which was the restricted 0–1 score, still showed very low reliability, whereas the reliabilities of the other three scores (adjustment, absolute adjustment, 0–1 score) were even negative. This result means that none of the four scores can be seen as a more reliable measurement of anchoring susceptibility than the others because none of them were actually reliable.

There are several possible explanations for these results. Certain characteristics of the present study could have led to these particularly low reliabilities. In particular, the high heterogeneity of the anchoring items is a likely reason for an inconsistently strong anchoring effect across the various items for one person. Because the items came from different domains (e.g., geography, history), prior knowledge could have impacted the anchoring effect sizes. When people have absolutely no idea about the true value of an item, they might just stick close to the anchor number, resulting in a seemingly stronger anchoring effect. When people know the exact value of an item, anchoring appears to vanish for this item. The most interesting case of anchoring is when people have to estimate the correct value on the basis of a vague idea of the possible scope. In the present study, after completing the anchoring task, participants had to indicate for every item whether they guessed the true value (i.e., they had no idea what the true value was), estimated it (they had a vague idea about what the true value was), or knew it. In the last case, the item was excluded from further analyses. As Table 2 shows only small to medium effect sizes for prior knowledge, adjustment strength does not seem to depend on previous knowledge about different items, meaning that the low reliabilities cannot be explained by prior knowledge. However, the role that prior knowledge about the items plays in an anchoring task should be further clarified in future research.

The range of plausible values is another factor that varied across items and could have led to different anchoring effect sizes and therefore to the low reliability of the susceptibility to anchoring scores. As an example: Relying on common sense, the average height of a woman in Germany can lie only within a relatively small range, that is, between about 160 cm to 170 cm. The smallest distance between Russia and the US—without previously knowing that the U.S. state of Alaska is so close to Russia—could also be estimated to be around several thousand kilometres, even though the true value is only 4 km, so even the high anchor was “only” 7 km. Differences between the estimate and the anchor can therefore be comparatively high in certain items, resulting in varying anchoring scores for one person and therefore in the low reliabilities of the anchoring susceptibility scores. In this respect, item heterogeneity can be seen as a limitation of this research.

Overall, for the present study, we chose to present a variety of anchoring items as part of our aim to obtain a valid measurement of anchoring. By not measuring estimation tendencies, which are based on the type of item, we limited the extent to which we artificially boosted the reliabilities of the anchoring scores. The low reliabilities in this study indicate that in studies in which the reliabilities of the anchoring susceptibility measurements are acceptable, the validities deserve particular scrutiny. In fact, recent examinations from the OpAQ data set, which already contains more than 50,000 anchoring trials, have shown that reliability was either low as it is in most tasks, or if not, the anchoring scores most likely lacked validity (Röseler et al., 2024).

### Explanation for the Reliability Problem

The most plausible reason for the low reliabilities of the anchoring scores found in the present research as well as in more comprehensive meta-analyses is that there are no conditions under which anchoring susceptibility can be measured reliably. This can be explained either by the methodology employed in anchoring experiments or by the idea that anchoring susceptibility is not a stable personality trait. In this latter case, anchoring can be seen as an effect that underlies a range of situational factors but not as a general person-dependent susceptibility.

### Limitations

Participants in this study were recruited via platforms that are especially likely to recruit other researchers as participants (occupation was not recorded in the demographic questions). A more diverse sample would improve the representativeness and generalizability of the results. In these platforms, because participation in surveys is rewarded with points needed to gather participants for one’s own research, the motivation of the participants can also be questioned. The motivation to give correct answers and the motivation to avoid being influenced by the anchor were recorded. Figure 2 illustrates that participants generally indicated rather high motivation, and the motivation to give correct estimates was slightly higher than the motivation to avoid being influenced by the anchor. Exploratory analyses showed no systematic influence of motivation on the reliabilities of the anchoring measurements, as shown in Table 3. However, it should be considered that the results on the motivation scales could be influenced by social desirability. This is also the case for the replies to the control question about whether participants used external sources.

As already described, the choice of items and their heterogeneity can be seen as a strength of this study but also as a weakness in some ways. Some items are probably more suited than others for measuring anchoring and susceptibility to it.

Later exclusions of one of the original 18 items as well as of one of the original 79 survey trials are further limitations of the present research, as these are deviations from the preregistration and led to a minor decrease in power. Statistical analyses including all the original items and participants did not show any differences in the significance of the hypothesis tests.

### Conclusion and Further Research

The present study indicates that anchoring susceptibility cannot be measured reliably, independent of the type of the currently existing anchoring scores. The results suggest that the methodology of measuring anchoring susceptibility should be further examined and improved. If it turns out that this is not possible, existing theories that are based on the assumption that anchoring susceptibility is a stable individual parameter should be called into question. In future research, the psychological mechanisms underlying anchoring and its strength should be investigated by taking a closer look at person-independent factors rather than individual parameters.

## Supplementary Materials

For this article, the following Supplementary Materials are available:

- Data. (Röseler et al., 2022)
- Code. (Röseler et al., 2022)
- Study materials. (Röseler et al., 2022)
- Preregistration. (Weber et al., 2021)

## Data Availability

For this article, data, code and study materials are available at Röseler et al. (2022). The preregistration is available at Weber et al. (2021)
